# Chest Computed Tomography and Lung Ultrasound Findings in COVID-19 Pneumonia: A Pocket Review for Non-radiologists

**DOI:** 10.3389/fmed.2020.00375

**Published:** 2020-06-26

**Authors:** Davide Pata, Piero Valentini, Cristina De Rose, Rita De Santis, Rosa Morello, Danilo Buonsenso

**Affiliations:** ^1^Istituto di Pediatria, Università Cattolica del Sacro Cuore, Rome, Italy; ^2^Dipartimento Scienze della salute della donna, del bambino e di sanità pubblica, Fondazione Policlinico Universitario Agostino Gemelli IRCCS, Rome, Italy; ^3^Istituto di Microbiologia, Università Cattolica del Sacro Cuore, Rome, Italy

**Keywords:** COVID-19, chest computed tomography, lung ultrasound, SARS-CoV-2, diagnosis

## Abstract

COVID-19 is an infectious disease that has quickly spread worldwide, causing a pandemic. The main clinical manifestation is pneumonia. The most important test for the diagnosis is represented by RT-PCR, but, given the limited sensitivity, further radiological examinations are necessary. We reviewed the literature to highlight the typical manifestations and advantages of chest computed tomography and lung ultrasound in COVID-19 pneumonia in order to assist clinical researchers in the management of this disease.

## Introduction

COVID-19 (COronaVIrus Disease 2019) is an infectious disease caused by a novel Coronavirus called SARS-CoV-2. Although initially described in Wuhan (1), the disease quickly spread worldwide, and the World Health Organization (WHO) defined COVID-19 as a pandemic on March 11th, 2020^1^. On April 8th, 1,353,361 cases had been confirmed from all over the world (2).

The incubation period is between 1 and 14 days and averages 3–7 days (3). The common and mild symptoms are fever, fatigue, cough, pharyngitis, myalgia, arthralgia, anosmia, and dysgeusia (4–6). However, the most important clinical manifestation of COVID-19 is pneumonia, which may be complicated by acute respiratory distress syndrome (ARDS), leading, in some cases, to acute respiratory failure and exitus (7, 8).

The main test for the diagnosis of SARS-CoV-2 infection is the real-time reverse transcription polymerase chain reaction (RT-PCR), which is performed by nasopharyngeal swab. However, given the high rate of false negative results (9), several authors suggested the routine use of chest computed tomography in case of COVID-19 suspicion (CT) (10, 11). Fang et al. reported that CT scans have a sensitivity of 98% (12). However, Raptis et al. pointed out a series of bias errors and assumed a lower sensitivity (13). Another study reported that CT had limited sensitivity especially in the early stages of the disease: in the first 2 days after the onset of symptoms, 56% of patients had normal findings, and chest CT can therefore not be used to exclude SARS-CoV-2 infection (14). In addition, this test was used in subsequent monitoring (15).

Recent studies established that Lung Ultrasound (LUS) is a reliable technique in the diagnosis of lung diseases (16–19), even during pregnancy (20). As a result, a number of manuscripts were produced on the use of pulmonary Lung Ultrasound (LUS) during the COVID-19 epidemic.

Therefore, we performed a literature review on the use of CT and LUS in COVID-19 pneumonia in order to assist non-radiologists involved in the forefront of COVID-19.

## CT Findings

In their work, Shi et al. reported that all patients had pathological CT scans. The abnormalities can be seen in all lung segments and their number is directly related to disease severity. The most frequently reported distribution pattern is bilateral lung involvement (79%), while peripheral and diffuse distributions are rarer, 54 and 44%, respectively (21).

**Figure 1:**
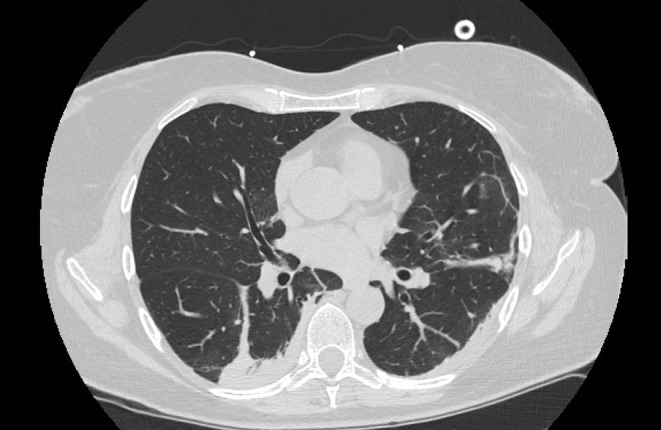
Typical CT findings in patients with COVID-19 pneumonia. Peripheral ground glass lesion with consolidations and bronchiectasis.

**Figure 2:**
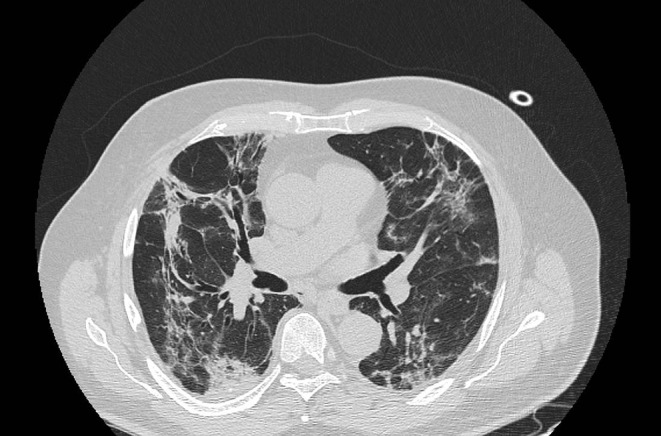
Typical CT findings in patients with COVID-19 pneumonia. Consolidations and GGO, associated with bronchiectasis and crazy paving pattern.

The typical CT pattern is characterized by bilateral distribution of ground glass opacities (GGO) with or without consolidations in posterior and peripheral lung fields (22, 23) (Figures 1, 2):

- ***GGO*** refers to areas of misty pulmonary opacity with conservation of parenchymal architecture, caused by the thickening of the alveolar septa due the inflammatory process (24). In COVID-19 pneumonia, unilateral or bilateral GGO are the most common CT findings (25–28)
- ***Reticular pattern*** refers to the presence of several linear opacities that give the appearance of a network. It is due to the interstitial thickening (24). Reticular pattern is the most frequent pattern in COVID-19 patients after GGO (21, 26, 29)
- ***Crazy paving pattern*** is defined as thickened interlobular septa superimposed on an area of GGO and is due to alveolar proteinosis (24). It indicates the transition to a progressive stage of COVID-19 pneumonia (30)
- ***Consolidation*** is an area of increased density with the elimination of normal lung parenchymal architecture. Air is replaced by fluids or cells, while vascular structures and bronchial walls are no longer recognizable (24). Consolidation is usually described in COVID-19 patients (28–30) and is another sign of disease progression (30)
- ***Pleural abnormalities***, such as thickening and effusion, are less frequently described in COVID-19 pneumonia and could be a poor prognostic sign (21)
- ***Airway abnormalities*** include bronchiectasis and bronchial wall thickening. They are due to inflammatory damage (24) and are described in the most severe pneumonia (21)
- ***Air bronchogram*** is the direct visualization of an air-filled bronchus in the context of an opacity (24). Indeed, in COVID-19 the bronchus is filled of highly viscous mucus instead of air (31)
- ***Fibrosis*** is defined as the replacement of normal tissue into scar tissue. According to some authors fibrosis could indicate a recovery (25). Instead, others described it as a poor prognostic sign (30)
- ***Lymphadenopathy*** is an inflammation of mediastinal lymph nodes. Although rare, it is reported in more severe pneumonia or in bacterial superinfection (28)
- Other rarely associated findings are ***nodules***, small regular or irregular opacity (25), ***halo sign***, a nodule encircled by hazy area (32), and ***subpleural*** ***curvilinear line*** (29).

Pan et al. correlated the typical CT findings with the stage of the disease (30).

1. Early stage: it occurs in the first 4 days after the onset of the disease and the typical CT finding is GGO. It can be unilateral or bilateral in subpleural localization, and it is more frequently described in the lower lobes;
2. Progressive stage: between 5 and 8 days after the onset of the pneumonia, it is characterized by the extension of GGO and by the appearance of crazy paving pattern and consolidation;
3. Peak stage: it represents the evolution of the previous stage, and consolidation became the main CT finding;
4. Absorption stage: it occurs more than 2 weeks after the onset of the infection. The patient moves toward recovery and the findings listed above disappear with the exception of GGO.

## LUS Findings

In order to limit the subjectivity of the exam and to obtain comparable data, Soldati et al. described a standardized protocol (33). It requires the examiner to analyze 14 intercostal regions for 10 seconds, with the focal point set on the pleural line. A portable convex probe (3.5 mHz), connected wirelessly with a tablet, should be used. A first operator performs the examination, while a second operator placed at a safe distance takes care of the image management (34). In fact, despite the processing of lower quality images compared to other devices, this mode is more easily performed in the current epidemic scenario. It allows to perform the exam bedside, avoiding the movement of unstable patients, and protects operators from possible contagion using disposable plastic covers for the device, preventing any subsequent spread of the outbreak (35).

**Figure 3:**
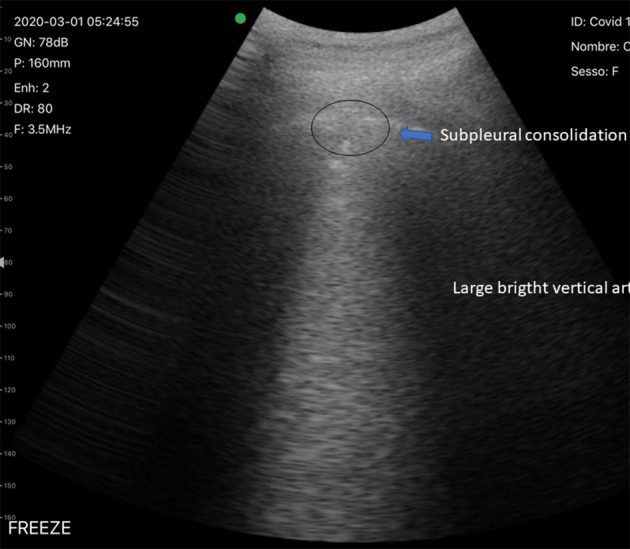
Typical LUS findings in patients with COVID-19 pneumonia. Subpleural consolidation and vertical artifact.

**Figure 4:**
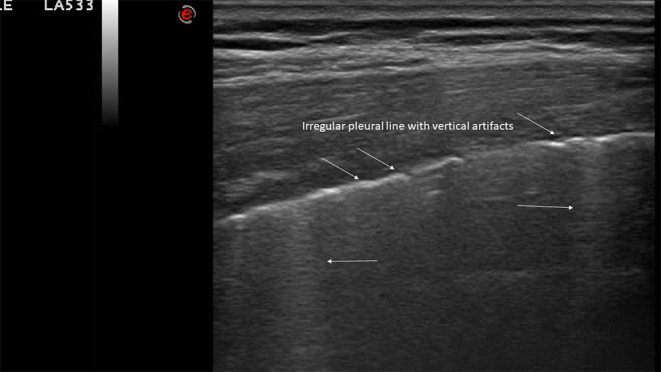
Typical LUS findings in patients with COVID-19 pneumonia. Irregular pleural line with vertical artifacts.

Typical LUS findings can be found in all lung fields, although bilateral posterior/lateral ones are more frequently involved (36) (Figures 3, 4):

- ***B-lines***: vertical artifacts generated by the variation of the acoustic impedance due to the inflammatory process (18, 37); typically, vertical artifacts in COVID-19 patients are long, touch the bottom of the ultrasound screen, and are bright and thick;
- ***White lung***: regions of white areas with the absence of A-lines (horizontal and hyperechoic lines due to the normal reflection of the ultrasound beam) and vertical artifacts, which correspond to increased density of the lung parenchyma (18);
- ***Subpleural consolidations***: irregular hypoechoic areas, indicating a collapsed lung or atelectasis (18, 37);
- ***Pleural line irregularities***, such as thickening or interruptions, caused by the replacement of air with blood, pus, and fibrin according to Huang (36);
- ***Air bronchograms*** and ***pleural effusions*** are very rare and unusual, and their presence should first let the clinician thinking other diagnosis or superinfections (38).

LUS findings are related to the extent of lung injury (39). In the early stages, the lesions described are irregular vertical artifacts (B-lines) with small regions of white lung. In the intermediate stages, these lesions extend over a larger lung surface. In case of respiratory failure, subpleural consolidations are reported in a gravitational position associated with air bronchograms and large regions of white lung (36, 39, 40). Furthermore, the diagnostic efficacy of LUS is high especially for severe patients (41).

Consequently, in order to allow comparing the severity of COVID-19 pneumonia of different patients, limiting the subjectivity and the operator-dependence of the exam, Soldati et al. proposed a **LUS Score of Severity of COVID-19 Related Findings** **(****33****):**

- Score 0: normal LUS pattern characterized by regular pleural line and A-lines;
- Score 1: vertical artifacts are described. The pleural line appears indented with several B-lines;
- Score 2: a broken pleural line with dark and white consolidation areas are described;
- Score 3: large regions of white lung.

However, LUS cannot be considered the gold standard for diagnosis. A limitation is that this exam cannot describe the deep lung abnormalities, since ultrasound is blocked by the presence of air. Conversely, LUS is very sensitive in detecting in small peripheral lesions and pleural effusion (36).

The typical LUS pattern of COVID-19 pneumonia is the patchy and bilateral distribution of the main lesions (42). In agreement with a group of international experts (43), during the current epidemiological scenario, the described LUS patterns in the context of fever and/or respiratory symptoms, reduced lymphocytes, and increased levels of protein C-reactive, LDH, and ferritin, are suggestive of COVID-19 pneumonia.

## Discussion

Six months after the first description of COVID-19 in China, the pandemic is still ongoing, and several countries are still facing the peak of the disease. Although there are still several questions to be answered regarding SARS-CoV-2 infection, the role of imaging in this pandemic is fundamental. While nasopharyngeal swabs can only diagnose the infection, CT scanning and LUS are both necessary to diagnose disease (COVID-19), even in case of negative microbiological results, since up to 30% of COVID-19 cases have false negative nasopharyngeal swabs. Both CT and LUS have high sensitivity to diagnose COVID-19 pneumonia and each of them has specific advantages. CT scanning is the gold standard and can easily diagnose also COVID-19 related complications, including thrombotic-hemorrhagic events, not rare in COVID-19 patients. LUS is easy to perform, can be performed at bedside or even at home, and would be a feasible option also in low to middle-income countries (44). Both tests can be used as triage tools to assess those with pathological findings that would need hospitalization, allowing a proper use of the limited resources of the health systems worldwide. For these reasons, every healthcare workers should be aware of the main CT and LUS patterns of COVID-19.

## Conclusion

In our opinion, the use of LUS during the COVID-19 outbreak has many advantages over CT, such as the bedside execution, the need for fewer operators, and the possibility of performing it at home and thus avoiding hospitalization of patients and overcrowding of the hospital. It is also less expensive (therefore easier to obtain in a developing country) and does not use ionizing radiation. Also, LUS can be used to monitor patients requiring serial examinations and in the evaluation of pregnant women, since it avoids exposure of the fetus to radiation (45).

It is important to highlight that CT and LUS are not competitive but rather complementary tools that can be used in different settings to answer different clinical questions. CT scan offers a better and comprehensive view of the lung and can also help identify complications such as infarction, embolism, emphysema; therefore, CT scanning is always helpful in case of sudden worsening of clinical conditions or for an initial assessment of moderate to severe patients if feasible in the setting where the patient is evaluated. Conversely, LUS can be used as a first level exam during the first evaluation in the emergency department or even at home to distinguish low-risk from high-risk patients, as these would need second level exams or admission/discharge. It is useful for detecting small peripheral lesions and pleural effusion. In addition, pregnant women and children should be evaluated by LUS unless CT is considered necessary. Importantly, LUS should be preferred for follow-up and daily monitoring. Important aspects that need to be clarified are the sensitivity, the positive predictive value and the negative predictive value of the exam. Furthermore, the sharing of information and data on online platforms is essential in order to create an algorithm able to identify the typical CT and LUS findings of COVID-19 pneumonia, as already suggested by the researchers of the Italian Academy of Thoracic Ultrasound (ADET) (33).

## Author Contributions

DP and DB contributed conception and design of the paper and wrote the manuscript. PV, CD, RD, and RM collected the data and the articles. All authors contributed to manuscript revision, read and approved the submitted version.

## Conflict of Interest

The authors declare that the research was conducted in the absence of any commercial or financial relationships that could be construed as a potential conflict of interest.

## Footnotes

^1^Available online at: https://www.who.int/dg/speeches/detail/who-director-general-s-opening-remarks-at-the-media-briefing-on-covid-19-11-march-2020 (accessed April 04, 2020).
